# Acceptability and usability of oral fluid HCV self-testing among health-facility users from Brazil: a cross-sectional study of 685 participants

**DOI:** 10.1016/j.bjid.2025.104544

**Published:** 2025-05-23

**Authors:** Hugo Perazzo, Cristiane Villela-Nogueira, Maria K. Gomes, Andre Daher, Cristiane Siqueira-do-Valle, Ketiuce Zukeram, Ana Cristina G. Ferreira, Karen Cristine Tonini, Elton Carlos de Almeida, Sandra W. Cardoso, Beatriz Grinsztejn, Valdilea G. Veloso

**Affiliations:** a Fundação Oswaldo Cruz (FIOCRUZ), Instituto Nacional de Infectologia Evandro Chagas (INI), Rio de Janeiro, RJ, Brazil; b Universidade Federal do Rio de Janeiro (UFRJ), Hospital Universitário Clementino Fraga Filho (HUCFF), Faculdade de Medicina, Departamento de Medicina Interna, Rio de Janeiro, RJ, Brazil; c Uninivesidade Federal do Rio de Janeiro (UFRJ), Faculdade de Medicina, Departamento de Medicina em Atenção Primária à Saúde, Brazil; d Fundação Oswaldo Cruz (FIOCRUZ), Plataforma de Pesquisa Clínica FIOCRUZ, Vice-Presidency of Research and Biological Collections, Rio de Janeiro, Brazil; e Ministério da Saúde do Brasil, Departamento de HIV/AIDS, Tuberculose, Hepatites Virais e Infecções Sexualmente Transmissíveis (DATHI/SVSA/MS), Brasília, DF, Brazil

**Keywords:** Viral hepatitis, Screening, Feasibility, HCV testing, Self-testing

## Abstract

**Introduction and objectives:**

HCV Self-Testing (HCVST) can be used to uptake HCV testing. We aimed to evaluate the acceptability/usability and re-reading/re-testing agreement of oral fluid HCVST among health-facility users in the Primary Care Systemin Brazil.

**Materials and methods:**

Consecutive people aged 18‒79 years using the Primary Care System (PCS) from 04-July-2022 to 30-September-2022 were invited for this cross-sectional study. The professional use OraQuick® HCV Rapid Antibody Test was used as a HCVST prototype. Oral fluid HCVST was performed relying on a step-by-step video and written/pictorial instructions. Usability was assessed by observed errors and documented need of assistance by a Healthcare Worker (HCW). After HCVST, a second HCV test was performed by the HCW using the same test-kit. *Re*-reading and re-testing concordances were evaluated (Cohen’s kappa, κ). Post-testing participant’s perspectives were assessed.

**Results:**

685 participants (74.5% female; median age = 52 [IQR 39‒61] years, 52.5% with schooling ≤ 10 years) were included. Major observed errors [%(95%CI)] were incorrect sample collection [32.8% (29.4‒36.5)] and wrong placing the test device in the tube [15.0% (12.6‒17.9)]. A total of 35.6% (95% CI 32.1‒39.3) of participants needed assistance in at least one step of HCVST. *Re*-reading and re-testing agreements were 95.2% (κ = 0.56) and 99.7% (κ = 0.67; *n* = 626 excluding invalid tests), respectively. After HCVST, 93% felt safe, 99% would be willing to test again, and 99% would recommend HCVST. Most participants rated the HCVST experience as easy (73%) or very easy (24%).

**Conclusion:**

Oral-fluid HCVST was feasible and well-accepted among users of the PCS in Brazil. HCVST can be an alternative to scale-up HCV testing.

## Introduction

Hepatitis C Virus (HCV) infection is a major public health problem, since approximatively 58 million individuals have been living with chronic hepatitis C worldwide.^1^ The HCV cascade of care remains unsatisfactory in many countries, especially due to a relatively low rates of facility-based HCV testing and to several barriers from screening to HCV treatment.^2^ Additionally, the number of people tested for HCV infection dramatically decreased in 2020/2021 due to the COVID-19 pandemic, resulting in a reduction in the number of HCV treatment started. Hepatitis Elimination Programmes in several countries were disrupted due to lockdowns combined with over-burdened healthcare systems during the pandemic.^3^

Self-testing is an innovative strategy in which people can collect their own specimen and perform a test themselves. Currently, HIV self-testing (HIVST) has been recommended by the World Health Organization (WHO) as an alternative for HIV testing in several countries.^4^ Similarly, HCV Self-Testing (HCVST) can be a strategy to scale up HCV testing and population knowledge about their need to seek for specific care. WHO has been recommending HCVST as an additional approach to facility-based HCV testing. However, this WHO guidance suggests that use of HCVST should be adapted to local context and that this approach must be followed by HCV infection confirmation (HCV-RNA) and linkage-to-treatment.^5^ Recently, the first oral fluid HCVST test was pre-qualified by WHO (https://www.who.int/news/item/10–07–2024-who-prequalifies-the-first-self-test-for-hepatitis-c-virus). However, few studies have evaluated feasibility of HCVST, especially in large sample size of general population from Western countries. This study aimed to evaluate acceptability, usability, re-reading/re-testing agreement and participant’s perspectives post-HCVST among health-facility users in the Primary Care System in Rio de Janeiro (Brazil).

## Material and methods

### Study design and population

This cross-sectional study was conducted in a Basic Health Unit (*Clínica da Família Felippe Cardoso*) located in a poor region (*Complexo da Penha*) that is composed by 13 slums (*favelas*) with more than 40,000 habitants in Rio de Janeiro (Brazil). A campaign to encourage facility-based fingerstick HCV Rapid Test (RT) was implemented in this Basic Health Unit in July/2022. Consecutive people aged 18‒79 years seeking for HCV RT (SD Bioline® HCV, Abbott Laboratories, Chicago, Illinois, USA) in the Basic Health Unit from 04-July-2022 to 30-Sept-2022 were invited to participate in this study. Exclusion criteria were eating or drinking less than 15 mins prior to testing, use of oral care products 30 mins prior to testing or refusing to perform HCVST. This study was coordinated by the Evandro Chagas National Institute of Infectious Diseases from the Oswaldo Cruz Foundation (INI/FIOCRUZ) with support of the Health Department from the Municipality of Rio de Janeiro (*Secretaria de Saúde do Município do Rio de Janeiro*, SMS/RJ). The study protocol was approved by the Institutional Review Board (IRB) from INI/FIOCRUZ (IRB 42,225,821.9.1001.5262), and from SMS/RJ (IRB 42,225,821.9.3004.5279). All participants signed an informed consent prior to study enrollment.

### Pre-testing interview

Socio-demographic and clinical records included age, sex at birth, self-reported skin color, years of study, employment status and self-reported presence of type-2 diabetes, blood hypertension and HIV infection. Participants were interviewed for risk factors for HCV infection (blood transfusion, former of current inject drug use, tattoo/piercing and hemodialysis). Additionally, sexual behavior [number of different sexual partners in the last 6-months, condomless sex and homosexual intercourse] was assessed by a trained investigator. All participants of this study had fingerstick HCV SD Bioline® (Abbott, Chicago, Illinois, USA), an immunochromatographic Rapid Test (RT) for the qualitative detection of antibodies specific to HCV, as part of the study procedures. Data were entered in electronic forms of REDCap (Research Electronic Data Capture, https://www.project-redcap.org/).^6^

### HCVST procedure, re-reading/re-testing agreement and post-testing perspectives

Prototype self-testing kits that included a professional use OraQuick® HCV Rapid Antibody Test (OraSure, USA), a plastic stand and Instructions For Use (IFU) adapted for self-testing and approved by the test manufacturer were used for this study. A video with step-by-step instructions to perform and to interpret HCVST results was provided in a tablet for all participants. Additionally, high-quality A3 size printed IFU including written/pictorial instructions in local language on how to perform HCVST and how to interpret HCVST results was available in the room where participants should perform HCVST (Supplementary Material). Participants performed the HCVST while being observed by a trained healthcare worker in private rooms (one healthcare worker per participant). The healthcare worker noted errors with self-testing steps according to a standardized checklist, while observing the participant complete the self-testing procedure. The HCVST checklist included questions on pre-testing steps (watching the step-by-step video, reading IFU, opening the pouch, organizing the material on the table for testing, placing the test tube in the plastic stand), testing steps (collection of oral fluid specimen and keeping time correctly) and test interpretation steps (interpreting the results correctly).

Participants were encouraged/oriented to perform the HCVST on their own without assistance. However, the healthcare worker could provide assistance if requested by the participant. This need for assistance was noted by the healthcare worker as part of the assessment of the feasibility of oral fluid HCVST in this sample. Additionally, the healthcare worker noted which steps the participants required assistance during the HCVST procedure. HCVST results were first read and interpreted by the participant, and then the same results were read and interpreted by the healthcare worker. The interpretation of HCVST results by the participant and the healthcare worker were recorded in electronic forms. Results from HCVST were not used for clinical decision making, since those kits were used as Research-Use-Only (RUO). After interpretation of HCVST, all participants were tested by the healthcare worker with a second test, the OraQuick® HCV Rapid Antibody Test for professional use. Results from this second test were read and interpreted by the healthcare worker, communicated to the participants, and recorded in the electronic form. Results from the professional use of the OraQuick® HCV Rapid Antibody Test and/or the fingerstick HCV Bioline® were used for clinical decision. Post-test counselling was provided to all participants, and those with a positive HCV test were linked to confirmatory HCV-RNA testing and HCV treatment at INI/FIOCRUZ.

Finally, participants were interviewed by the healthcare worker to collect post-self-testing perspectives and preferences. Those questions included if participants felt safe performing HCVST; if they would be willing to recommend HCVST to a parent, friend, or sexual partner; if they would be willing to repeat HCVST and to perform a blood based HCVST. Additionally, participants were asked to categorize the overall experience and the interpretation of HCVST as “very easy”, “easy”, slightly difficult”, “difficult” or “very difficult".

### Statistical analysis

Descriptive analyses included reporting categorical variables as absolute (n) and relative frequency (%) and continuous variables as median (IQR). Groups were compared using Chi^2^-test for proportions and Mann-Whitney for quantitative variables. Proportion (95% Confidence Interval, 95% CI) of errors observed by the healthcare worker during the HCVST procedure and proportion of participants who needed assistance in at least for one step of self-testing were described. Participants who refused to perform HCVST were excluded from the concordance analyses. *Re*-reading agreement was calculated as the percentage of concordance between the participant’s and the healthcare worker’s interpretation of the HCVST performed by the participant. *Re*-testing agreement was calculated as the percentage of concordance between the participant’s interpretation of the HCVST result and the healthcare worker’s interpretation of the professional use test result, excluding invalid results. Additionally, re-reading and re-testing concordance were assessed using Cohen’s kappa. Acceptability was assessed through proportion of post-testing perspectives and experiences of the self-testing procedures. Statistical analyses were conducted using STATA for Windows (2017; StataCorp LP, College Station, TX, USA).

## Results

**Fig. 1:**
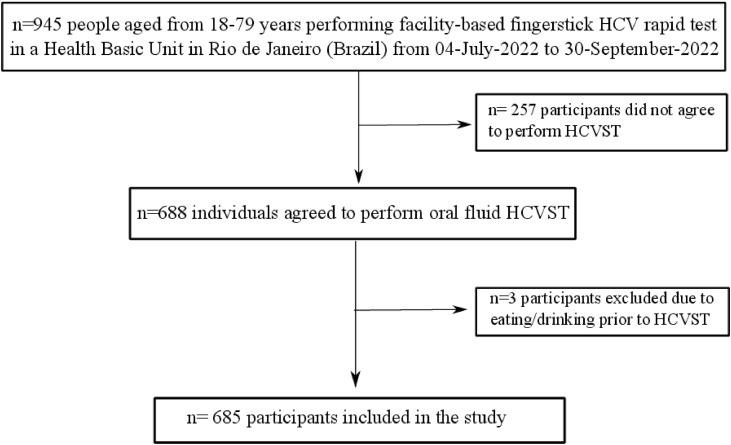
Flow-chart of study.

From 945 subjects who were seeking for finger-prick HCV RT during the study period, a total of 688 participants agreed to perform HCVST. Three participants were excluded due to eating or drinking 15 min before HCVST (Fig. 1). Therefore, 685 participants (74% female; median age of 52 [IQR 39‒61] years; 75% with self-report brown/mixed or black skin colour, 52.4% with schooling ≤ 10-years and 37.8% were unemployed) were included (Fig. 1). A total of 17.7% (*n* = 121) had type-2 diabetes and 41.9% (*n* = 287) had blood hypertension. Regarding risk factors for HCV infection, 6.1% (*n* = 42) had previous blood transfusion; 27.3% (*n* = 187) had tattoo or piercing and only 1.0% (*n* = 7) were People Who Inject Drugs (PWID). Additionally, the sexual risk of HCV transmission was low in this sample: 41.3% (*n* = 283) reported that they did not have sex 6 months prior to HCVST and those who had sex, 95.5% (*n* = 384/402) had 1‒2 sexual partners. Table 1 describes the socio-demographic characteristics of included participants. People who agreed to participate in the study (*n* = 688) were younger (age = 52 [IQR 39‒61] vs. 54 [44‒63], *p* = 0.028) and had a higher proportion of people with more than 10-years of schooling (47.2% vs. 32.7%, *p* < 0.001] compared to those who did not agree (*n* = 257) (Supplementary Table 1).

**Table 1:** Characteristics of participants included in the study.

	**All (*n* = 685)**
**Socio-demographic characteristics**	
Female sex at birth^a^	510 (74.5)
Age, years^b^	52 (39‒61)
Skin color^a^	
White	167 (24.4)
Black	168 (24.5)
Brown (mixed)	346 (50.5)
Other	4 (0.5)
Schooling^a^	
< 10-years	359 (52.4)
≥ 10-years	325 (47.5)
Preferred not to answer	1 (0.1)
Employment status^a^	
Formally employee	151 (22.0)
Informally employee	60 (8.8)
Freelance work	56 (8.2)
Unemployed	259 (37.8)
Retired	134 (19.6)
Other/ preferred not to answer	25 (3.6)
**Clinical features**^a^	
Type-2 diabetes	121 (17.7)
Blood hypertension	287 (41.9)
HIV infection	7 (1.0)
**Self-reported exposures to HCV risk factors**^a^	
Previous blood transfusion	42 (6.1)
Formal or current inject drug use	7 (1.0)
Tattoo or piercing	187 (27.3)
Formal or current haemodialysis	3 (0.4)
**Sexual behaviour in the last 6-months**^a^	
Sex intercourse in the last 6-months	402 (58.7)
Condomless sex^c^	
Yes, in all sex intercourses	296 (73.6)
Yes, but not in all sex intercourses	45 (11.2)
No	61 (15.2)
Homosexual sex intercourse^c^	19 (4.7)
Number of sex partners^c^	
1‒2 partners	384 (95.5)
3‒5 partners	14 (3.5)
≥ 6partners	4 (1)

Data expressed as n (%)^a^ or median (IQR)^b^.

^c^ Proportion of those who had sex intercourse in the last 6-months.

### Errors and assistance needed during hcvst

All 685 participants completed the HCVST process. Table 2 reports the proportion (95% CI) of observed errors and assistance provided during HCVST. In pre-testing procedures, 13.8% (11.5‒16.7) of participants did not watch the step-by-step video and 9.5% (7.5‒11.9) did not read the instructions. The healthcare worker observed errors for opening the package (4.4% [95% CI 3.1‒6.2]), for organizing the material (5.4% [95% CI 3.9‒7.4]) and for placing the tube into the stand (8.6% [6.7‒11.0]). The major proportion (95% CI) of observed errors were collecting the sample incorrectly (32.8% [29.4‒36.5]) and wrong placing of the test device in the test tube after sample collection (15.0% [12.6‒17.9]) Additionally, improper timekeeping for reading the test result was observed by the healthcare worker in 7.0% (95% CI 5.3‒9.2). Regarding the need for assistance, a total of 35.6% (95% CI 32.1‒39.3) of participants required assistance in at least one step to perform HCVST. Except for a significant higher proportion of correct timekeeping (94.5% vs. 88.1%, *p* = 0.005), there was no difference in proportion of other observed errors and assistance provided in at least one step in participants who read IFU and who saw the step-by-step video (*n* = 526) compared to those who did not (*n* = 159) (Supplementary Table 2).

**Table 2:** Observed errors and need of assistance provided by the healthcare worker during oral fluid HCV self-testing from 685 users of the Primary Care System in Rio de Janeiro.

	**All (*n* = 685)**	
	**n**	**% [95% CI]**
**Errors observed in pre-testing activities**		
Participants who did not watch the step-by-step video before HCVST	95	13.8 [11.5‒16.7]
Participants who did not read IFU before HCVST	64	9.5 [7.5‒11.9]
**Errors observed during HCV self-testing**		
Incorrect manipulation to collect oral fluid	225	32.8 [29.4‒36.5]
Wrong placing of the test device into the test tube	103	15.0 [12.6‒17.9]]
Error to place the tube into the plastic stand	59	8.6 [6.7‒11.0]
Improper timekeeping for reading results	48	7.0 [5.3‒9.2]
Error to organize the material on the table for HCVST	37	5.4 [3.9‒7.4]
Error to open the package	30	4.4 [3.1‒6.2]
**Assistance provided during HCV self-testing**		
Assistance requested for at least one step during HCVST	244	35.6 [32.1‒39.3]

CI, Confidence Interval; IFU, Instructions-For-Use; HCVST, HCV Self-Testing.

### *Re*-reading and re-testing concordance

Despite errors observed during HCVST, the re-reading and re-testing agreement were high (Table 3). A total of three participants refused to report their self-test results and were excluded from the concordance analyses. For re-reading concordance (*n* = 682), the agreement between the participant’s and the healthcare worker’s interpretation of the HCVST results was 95.2% with a Cohen’s Kappa of 0.56. A total of 4.3% (*n* = 29) of HCVST were interpreted as invalid by the participant but as negative by the healthcare worker. Additionally, a single HCVST (0.2%) was interpreted as invalid by the participant and positive by the healthcare worker and 20 self-tests (2.9%) were considered as invalid by both. Regarding re-testing concordance, the agreement of the participant’s interpretation of HCVST and the result of the HCV test performed by the healthcare worker (professional use) excluding invalid tests (*n* = 53; a total of 30 HCVST informed by participants; 20 HCVST informed by both participant and healthcare worker; and 3 invalid tests of the professional use of oral fluid HCV test) was 99.7% with a Cohen’s Kappa of 0.67. In sensitivity analyses, re-reading (κ = 0.60 vs. 0.48) and re-testing agreements (κ = 1.00 vs. 0.66) were better in people aged < 60 years compared to elderly participants (≥ 60-years) (Table 4, Table 5). On the other hand, there were no significant differences in re-reading (κ = 0.58 vs. 0.54) or re-testing agreement (κ = 0.67 vs. 0.67) between people with schooling lower than 10-years compared to those with schooling ≥ 10-years (Table 4, Table 5). The concordance between participant’s interpretation of oral fluid HCVST and fingerstick HCV RT was 98.9% with a Cohen’s kappa of 0.57.

**Table 3:** Assessment of re-reading and re-testing concordance.

	***Re*-reading by healthcare worker**^a^			
**Participant assessment**	**Negative**	**Positive**	**Invalid**	**Total**
Negative	627	2	1	630
Positive	0	2	0	2
Invalid	29	1	20	50
Total	656	5	21	682
***Re*-reading agreement = 95.2%; Cohen's kappa = 0.56**				

	***Re*-testing by healthcare worker**^b^			
**Participant assessment**	**Negative**	**Positive**	**Invalid**	**Total**
Negative	622	2	‒	624
Positive	0	2	‒	2
Total	622	4	‒	626
***Re*-testing agreement = 99.7%; Cohen's kappa = 0.67**				

a The results of the HCV self-tests (OraQuick® HCV test) were reported by participants and re-assessed by a healthcare worker; *n* = 3 participants refused to inform HCV self-test result.

b The results of the self-tests (OraQuick® HCV test) reported by participants were compared to the results of oral fluid HCV test performed by a healthcare worker using a similar one-use-only kit (OraQuick® HCV test). Invalid tests informed by participants (*n* = 50) or by healthcare worker (*n* = 3) were excluded; 3 participants refused to be tested by healthcare worker.

**Table 4:** Sensitivity analyses of re-reading concordance according to age (< 60-years vs. ≥ 60-years) and to schooling (< 10-years vs. ≥ 10-years of study)^a^.

**Analysis by age (*n* = 682)**				
	**Age < 60 years-old**			
**Participant assessment**	**Negative**	**Positive**	**Invalid**	**Total**
Negative	445	1	1	447
Positive	0	0	0	0
Invalid	18	0	16	34
Total	463	1	17	481
***Re*-reading agreement = 89.7%; Cohen's kappa = 0.60**				

	**Age ≥ 60 years-old**			
**Participant assessment**	**Negative**	**Positive**	**Invalid**	**Total**
Negative	182	1	0	183
Positive	0	2	0	2
Invalid	11	1	4	16
Total	193	4	4	201
***Re*-reading agreement = 93.5%; Cohen's kappa = 0.48**				

**Analysis by schooling (*n* = 682)**				
	**Schooling < 10 years of study**			
**Participant assessment**	**Negative**	**Positive**	**Invalid**	**Total**
Negative	329	0	1	330
Positive	0	1	0	1
Invalid	15	1	11	27
Total	344	2	12	358
***Re*-reading agreement = 88.8%; Cohen's kappa = 0.58**				

	**Schooling ≥ 10 years of study**			
**Participant assessment**	**Negative**	**Positive**	**Invalid**	**Total**
Negative	297	2	0	299
Positive	0	1	0	1
Invalid	14	0	9	23
Total	311	3	9	323
***Re*-reading agreement = 89.3%; Cohen's kappa = 0.54**				

a The results of the HCV self-tests (OraQuick® HCV test) were reported by participants and re-assessed by a healthcare worker; *n* = 3 participants refused to inform HCV self-test result.

**Table 5:** Sensitivity analyses of re-testing concordance excluding invalid tests according to age (< 60-years vs. ≥ 60-years) and to schooling (< 10-years vs. ≥ 10-years of study)^a^.

**Analysis by age (*n* = 682)**			
	**Age < 60 years-old**		
**Participant assessment**	**Negative**	**Positive**	**Total**
Negative	442	0	442
Positive	0	0	0
Total	442	0	442
***Re*-reading agreement = 100%, Cohen's kappa = 1.00**			

	**Age ≥ 60 years-old**		
**Participant assessment**	**Negative**	**Positive**	**Total**
Negative	180	2	182
Positive	0	2	2
Total	180	4	184
***Re*-reading agreement = 96.8%; Cohen's kappa = 0.66**			

**Analysis by schooling (*n* = 682)**			
	**Schooling < 10 years of study**		
**Participant assessment**	**Negative**	**Positive**	**Total**
Negative	326	1	327
Positive	0	1	1
Total	326	2	328
***Re*-reading agreement = 99.7%; Cohen's kappa = 0.67**			

	**Schooling ≥ 10 years of study**		
**Participant assessment**	**Negative**	**Positive**	**Total**
Negative	295	1	296
Positive	0	1	1
Total	295	2	297
*Re*-reading agreement = 99.7%; Cohen's kappa = 0.67			

a The results of the HCV self-tests (OraQuick® HCV test) were reported by participants and re-assessed by a healthcare worker; *n* = 3 participants refused to inform HCV self-test result.

A total of 8 participants (1.7%) were referred for HCV-RNA due to positive fingerstick HCV RT or professional use of oral fluid HCV test: both tests were positive in two participants; three people had positive fingerstick and negative oral fluid HCV tests; and three individuals had negative fingerstick and positive oral fluid test. Of them, 2 participants had detectable HCV-RNA; 4 had undetectable HCV viral load and 2 refused to perform this test. From those who had an undetectable HCV-RNA, two subjects reported previous HCV treatment (suggesting a probable sustained virological response).

### Post-testing perspectives

**Fig. 2:**
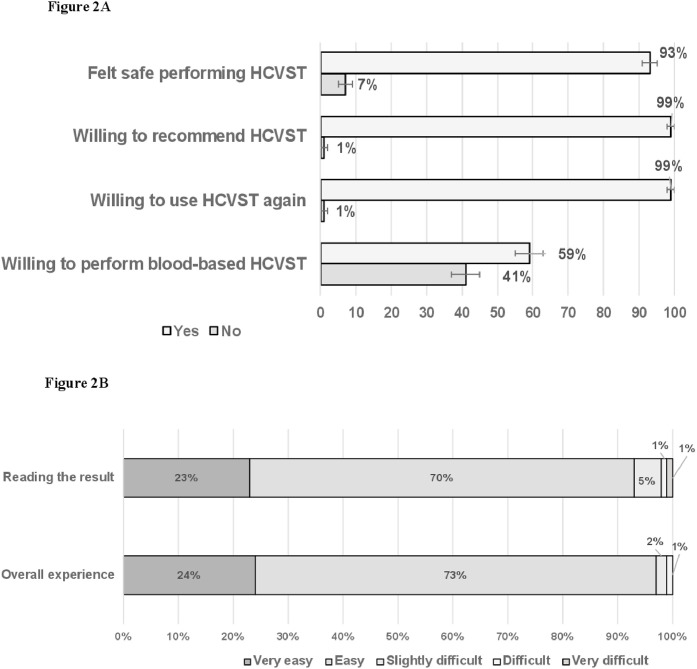
Overall participant’s perspectives post-HCV self-testing (A) and perception of difficult for reading results and performing HCVST (B).

In post-testing analyses, most people felt safe performing HCVST 93% (95% CI 91‒95) and most people would be willing to recommend HCVST for a parent, a friend, or a sexual partner 99% (95% CI 99‒100) and would be willing to repeat HCVST 99% (95% CI 98‒99). However, 41% (95% CI 37‒45) would not perform blood-based HCVST (Fig. 2A). Participants reported that the main advantages of HCVST would be the easiness (93%), the convenience (30%) or the privacy (29%) of HCVST. People reported that interpreting HCVST result was mostly easy 70% (95% CI 67‒73) or very-easy 23% (95% CI 20‒26). Additionally, 73% (95% CI 70‒77) and 24% (95% CI 21‒27) reported that the overall experience of HCVST was easy or very-easy, respectively (Fig. 2B).

## Discussion

This study highlighted the feasibility and re-reading/re-testing agreement of HCVST using oral-fluid kits in general population. Additionally, HCVST was very well accepted for HCV testing by participants from the Primary Care System from Rio de Janeiro (Brazil). Our findings have implications for validating HCVST as an useful tool to scale up HCV testing in strategies targeting the (micro) elimination of HCV by 2030.^7^

Few studies with limited sample size have described the acceptability and usability of HCVST using OraQuick® HCV Rapid Antibody Test. Those studies included mostly key populations and were conducted in different countries: people with chronic liver disease (*n* = 95) from USA,^8^ general population from Egypt (*n* = 116),^9^ PWID (*n* = 150) from Kenya,^10^ Men who have Sex with mMen (MSM) from China^11^ and MSM and/or PWID from Vietnam (*n* = 104 MSM and *n* = 105 PWID),^12^ and Georgia (*n* = 100 MSM and *n* = 100 PWID).^13^ A recent systematic review and meta-analysis that pooled data from those previous studies reported relatively high estimates for the usability and re-reading/re-testing agreement of oral fluid HCVST. Briefly, in this review the pooled proportion of people who correctly collected oral fluid sample collection was 87.2% (95% CI 76.0‒95.3) and the pooled proportion of people who performed HCVST without needing assistance in any step was 62.6% (95% CI 37.2‒84.8). Additionally, the pooled rate (95% CI) of re-reading 95.0% (91.5‒97.6) and re-testing agreement (94.4% [90.3‒97.5]) were high. Finally, most people would be willing to test again (pooled = 92.6% [95% CI 86.7‒97.0]) and would recommend HCVST to a friend/relative or sexual partner (pooled rate = 94.4% [95% CI 84.7‒99.6]).^14^ Most findings of the present study conducted in a large sample size among Primary Care System users in Brazil are aligned with the results from this previous systematic review/meta-analysis. We observed a similar proportion of people who needs assistance for self-testing, high rates of re-reading and re-testing agreement and high acceptance in post-testing questionnaires. On the other hand, we reported a higher proportion of people who incorrectly collected oral fluid sample 32.8% (95% CI 29.4‒36.5). This might be explained by the fact that most studies included in the systematic review were performed in key population who are more used to perform HIV self-testing than people attending the Primary Care System. Despite this relatively high proportion of incorrect sample collection and the need of assistance in our study, we observed a high re-reading and re-testing concordance for oral fluid HCV test.

In the present study, we observed that 41% (95% CI 37‒45) of participants would not perform blood based HCVST. Similarly, pooled data from the systematic review reported that 66.4% (95% CI 60.5‒72.1) would prefer oral-fluid rather than blood-based HCVST.^14^ Data on acceptability and usability of blood-based HCVST remain scare. Majam et al. reported a high usability of three HCVST fingerstick prototypes: CareStart® (AccessBio, Inc., Somerset, NJ, USA), SD Bioline® HCV (Abbott Rapid Diagnostics Ltd., Jena, Germany) and First Response® HCV Card Test (Premier Medical Corporation Pvt Ltd., Gujarat, India).^15^ Additionally, an ongoing protocol is comparing HCVST using an oral-fluid (OraQuick® HCV Rapid Antibody Test) and a blood-based (First Response® HCV) HCVST in key population from Malaysia (NCT04982718) .^16^

The main hypothesis is that HCVST might increase the number of people tested, diagnosed, and referred for treatment and HCV cure. However, this strategy might have a higher cost compared to the standard-of-care. A controlled-trial conducted in MSM from China reported that HCVST increased HCV testing in 60% to 70% compared to the standard-of-care (community-engaged information and recommendation for facility-based testing). Those authors reported a slightly higher cost of HCVST (US$ 1445 vs. US$ 1309), but a lower cost per person tested (U$ 49.83 vs. US$ 654.52) in MSM without HIV infection when compared to standard-of-care.^17^ Additionally, a recent Cost-Effectiveness Analysis (CEA) reported that the cost of using HCVST might be extremely variable according to the country/setting and this strategy seems to be more cost-effective in populations with high prevalence of HCV infection.^18^

Few challenges remain to be addressed for implementing HCVST. Difficulties with instructions and/or collecting samples can be barriers/challenges for self-testing for STIs including HCV infection.^19^ Videos using artificial intelligence can help to reduce errors. For the present study, we used a video with animations and an IFU printed with high-quality figures approved by the manufacturer. In post-testing survey, most people included in our study classified the IFU as comprehensive (99.5%) and judged that the video helped them to perform HCVST (99.2%). The best type of delivery service of HCVST remains unclear. There are few trials evaluating different types of kits distribution (secondary distributions, postal or peer delivery models) or the use of internet-technologies to uptake HCV testing using HCVST in Georgia (NCT04961723),^20^ Malaysia (NCT04982718)^16^ and Pakistan (NCT04971538) .^21^ A potential concern could be the misuse or harm of HCVST (such as coercive testing, violence or discrimination). Since direct evidence for HCVST remain scare, experts have been supporting the use of indirect evidence from HIV self-testing to support HCVST recommendations.^22^ Therefore, misuse of HCVST might be low since there were few cases of coercion (*n* = 4/13,267)^23^ and *n* = 0/1,06,3^24^ in large sample studies that assessed misuse of HIV self-testing. Additionally, the WHO HCVST guideline reported that there was no difference in intimate partner violence comparing HIVST to standard testing (RR = 0.92, 95% CI: 0.60–1.12; moderate certainty evidence) .^25^

The best way to implement HCVST in HCV Elimination Programs must be adapted considering local context. Additionally, the use of this tool to improve the HCV continuum of care must be combined with governmental willing, extensive investment in awareness of HCV infection, community outreach and a well-defined linkage to HCV infection confirmation and access to treatment. In Brazil, people can perform HCV rapid tests in facility-based basic health units, and procedures for HCV care, including HCV viral load and universal access to highly effective direct-acting agents, are available for free as part of the Brazilian Public Health System. Despite the availability of those tools and drugs for free, the HCV cascade of care remains unsatisfactory in Brazil.^26^ This might be related to barriers from HCV testing to start of treatment but also associated with a high proportion of undiagnosed HCV infection due to an unsatisfactory rate of HCV testing. HCVST can be a useful tool for scale up HCV testing. However, this strategy will not impact in the HCV cascade of care if there is a deficient post-testing service.

Our study has limitations. Firstly, this study might have had a selection bias as people who agreed to participate in the study were younger and had higher schooling than those who did not agree. Additionally, we acknowledge that we included middle-aged women with lower education level rather than general population. However, all consecutive individuals aged 18‒79 years who accepted to have fingerstick HCV RT from July to September 2022 were invited to participate in this study. Additionally, there were no additional significant differences in socio-demographics characteristics between those who agreed compared to those who did not agree to participate in this HCVST study (Supplementary Table 1). Secondly, we acknowledge that this study was conducted in a setting of relatively low prevalence of HCV infection. We mostly recruited aged women with a low sexual risk of HCV infection in our study. However, this reflexes the profile of people attending a primary care health unit of the Brazilian Public Health System.^27^ Moreover, the aim of this study was to assess the usability of HCVST rather than to evaluate the diagnostic value of HCVST kits or the prevalence of HCV infection. Thirdly, we used OraQuick® HCV Rapid Antibody Test kits for professional use adapted for HCVST. Currently, there is no WHO prequalified HCVST kit in the market. All previous studies also used oral fluid HCV kits approved for health professional testing that were re-packed for self-testing. In the present study HCVST were used as RUO and all participants had fingerstick rapid test HCV SD Bioline® and professional use of OraQuick® HCV Rapid Antibody Test for clinical decision-making. Finally, the lack of a one-to-one in-person demonstration on how to use the HCVST kit by a trained study staff might be considered as a limitation. However, we provided a written/pictorial IFU and a step-by-step video translated into the local language that were approved by the Brazilian Ministry of Health, by local IRBs and by the local representative of kit manufacturer to be used exclusively for this study. Additionally, participants performed HCVST supervised by a healthcare worker. People may have felt embarrassed by the presence of the healthcare worker, and this could have impacted our findings regarding the proportion of errors during the procedure. Further studies might be needed to provide evidence of feasibility of unsupervised oral fluid HCVST.

The main strength was the large sample size included in this study. There is few evidence of usability of HCVST among general population, especially in Western countries. Previous studies that assessed feasibility of oral fluid HCVST have included a limited sample, mostly key populations, from Eastern Europe and Asia-Pacific regions. Additionally, only a single study has evaluated usability of HCVST in 116 participants from the general population from Egypt.^9^ We evaluated the feasibility of oral fluid HCVST among 685 users of the Primary Care System in Brazil, the largest country in Latin America. Other strengths would be the observation of HCVST procedures by a trained investigator, the performance of a second oral fluid HCV test by a healthcare worker for re-testing agreement and the use of fingerstick HCV RT for clinical decision.

## Conclusion

In summary, oral fluid HCVST was very well-accepted, feasible and had high re-reading/re-testing agreement in people attending the Primary Care System in Rio de Janeiro (Brazil). However, challenges in collecting samples and a relatively high proportion of individuals requiring assistance can pose significant barriers to HCVST, particularly in real-world settings. The use of HCVST might be a game changer to increase the number of people tested, especially in countries with hard-to-reach populations. However, health authorities and policy makers from different countries and/or regions would need to evaluate the best way to implement HCVST in their HCV Elimination Programs. Future research, especially evaluation of unsupervised testing, implementation, cost-effectiveness studies and those assessing different types of HCVST distribution, are needed considering local context to support the use of HCVST as a public health strategy.

## Abbreviations

HCV, Hepatitis C-Virus; HIVST, HIV Self-Testing; WHO, World Health Organization; HCVST, HCV Self-Testing; INI/FIOCRUZ, Instituto Nacional de Infectologia Evandro Chagas da Fundação Oswaldo Cruz; SMS/RJ, *Secretaria de Saúde do Município do Rio de Janeiro*; IRB, Institutional Review Board; RT, Rapid Test; REDCap, Research Electronic Data Capture; IFU, Instructions For Use; RUO, Research Use Only; CI, Confidence Interval; PWID, People Who Inject Drugs; MSM, Men who have Sex with Men; CEA, Cost-Effectiveness Analysis.

## Ethics approval and consent to participate

The study protocol was approved by the Institutional Review Board (IRB) from INI/FIOCRUZ (IRB 42,225,821.9.1001.5262), and SMS/RJ (IRB 42,225,821.9.3004.5279). All participants signed an informed consent prior to enrollment in this study.

## Availability of data and materials

All data from the current study were based on published studies and reported in the manuscript, tables and supplementary material. In addition, data are available upon request to Hugo Perazzo, the corresponding author, from Evandro Chagas National Institute of Infectious Diseases (INI), Oswaldo Cruz Foundation (FIOCRUZ), Rio de Janeiro (RJ), Brazil.

## Funding

This work was supported by the 10.13039/501100004586Fundação Carlos Chagas Filho de Amparo à Pesquisa do Estado do Rio de Janeiro (FAPERJ) [grant number E-26/201.351/2021 and E-26/203.693/2021]; and the 10.13039/501100003593Conselho Nacional de Desenvolvimento Científico e Tecnológico (CNPq) [grant number 445957/2020-4]. The funders had no role in study design, data collection and analysis, decision to publish or preparation of the manuscript.

## CRediT authorship contribution statement

**Hugo Perazzo:** Conceptualization, Supervision, Formal analysis, Funding acquisition, Writing – original draft, Writing – review & editing. **Cristiane Villela-Nogueira:** Data curation, Investigation, Writing – review & editing. **Maria K. Gomes:** Data curation, Investigation, Writing – review & editing. **Andre Daher:** Project administration, Data curation, Investigation, Writing – review & editing. **Cristiane Siqueira-do-Valle:** Project administration, Data curation, Investigation, Writing – review & editing. **Ketiuce Zukeram:** Project administration, Data curation, Investigation, Writing – review & editing. **Ana Cristina G. Ferreira:** Supervision, Data curation, Writing – review & editing. **Karen Cristine Tonini:** Supervision, Data curation, Writing – review & editing. **Elton Carlos de Almeida:** Supervision, Data curation, Writing – review & editing. **Sandra W. Cardoso:** Supervision, Data curation, Writing – review & editing. **Beatriz Grinsztejn:** Conceptualization, Supervision, Investigation, Writing – review & editing. **Valdilea G. Veloso:** Conceptualization, Supervision, Investigation, Writing – review & editing.

## Conflicts of interest

The authors declare no conflicts of interest.

## Appendix Supplementary materials

Image, application 1
