# Supplementary material for: Acceptability and usability of oral fluid HCV self-testing among health-facility users from Brazil: a cross-sectional study of 685 participants
Source: Braz J Infect Dis. 2025 May 23;29(4):104544. doi: 10.1016/j.bjid.2025.104544 (PMC12151183; doi:10.1016/j.bjid.2025.104544)
Supplement: Supplementary file 1 [file mmc1.docx]

**BJID-D-24-00312_ Supplementary Material**

**Supplementary Material**

**Acceptability and usability of oral fluid HCV self-testing among health-facility users from Brazil: a cross-sectional study of 685 participants**

Hugo Perazzo; Cristiane Villela-Nogueira; Maria K. Gomes; Andre B. Daher; Cristiane Siqueira-do-Valle; Ketiuce Zukeram; Ana Cristina G. Ferreira; Karen Cristine Tonini; Elton Carlos de Almeida; Sandra W Cardoso; Beatriz Grinsztejn; Valdilea G Veloso

|  | **Page** |
| --- | --- |
| **Written/ pictorial instructions for use (IFU) used in the study.** | 1 |
| **Supplementary Table 1.** Study characteristics of those individuals who agreed compared to those who did not agree to participate in the study | 2 |
| **Supplementary Table 2.** Comparison of observed errors and assistance provided between participants who did and who did not read instructions for use and saw the step-by-step video before HCV self-testing | 3 |

**Written/ pictorial instructions for use (IFU) used in the study.**


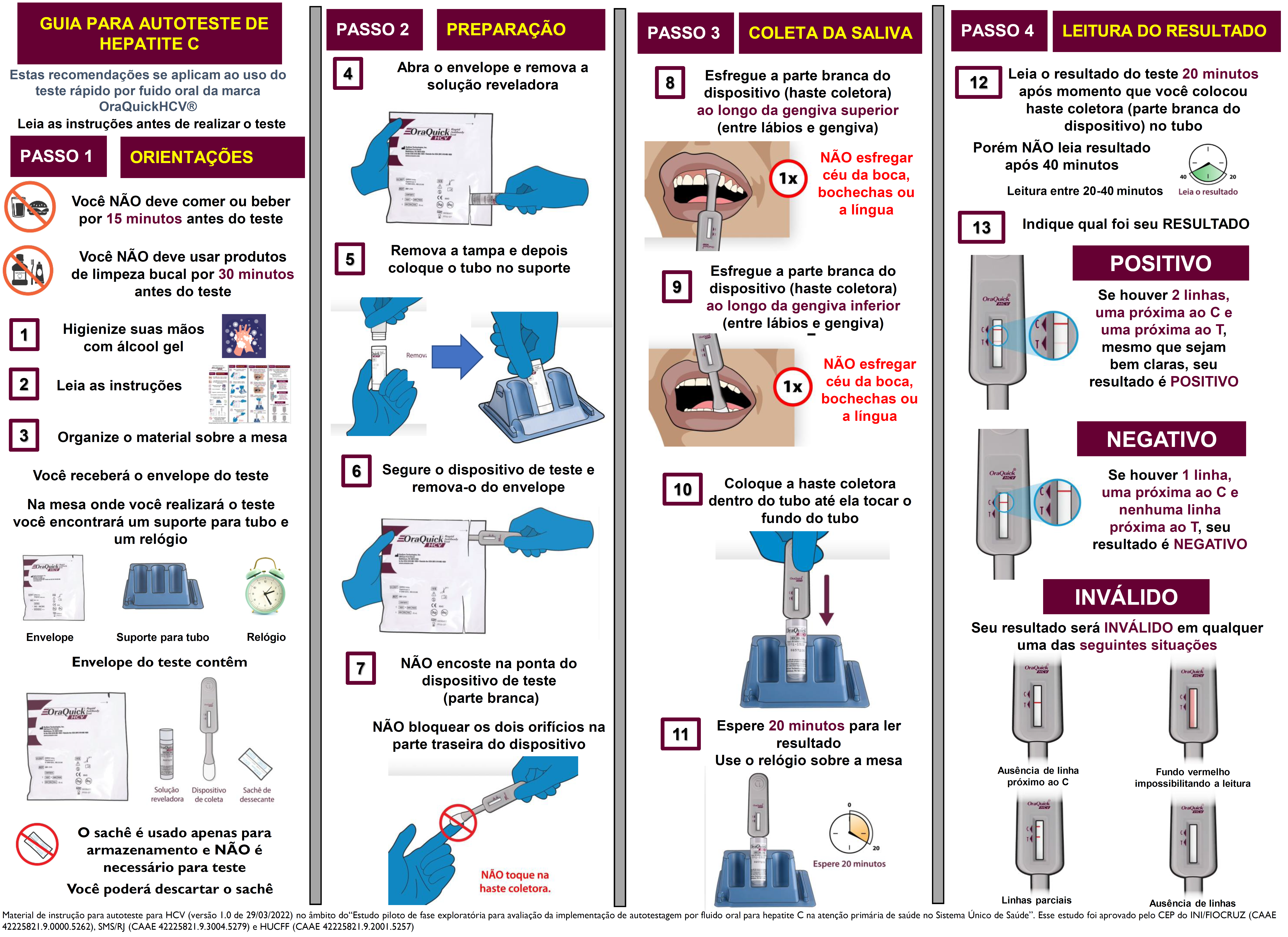


**Supplementary Table 1.** Study characteristics of those individuals who agreed compared to those who did not agree to participate in the study

|  | **People who did not agree (n=257)** | **People who agreed (n=688)** | **P value** |
| --- | --- | --- | --- |
| **Socio-demographic characteristics** | | | |
| Female sex at birth ^a^ | 190 (73.9) | 512 (74.4) | 0.88 |
| Age, years ^b^ | 54.0 (44.0, 63.0) | 52.0 (39.0, 61.0) | 0.028 |
| Skin color ^a^ |  |  | 0.40 |
| White | 58 (22.6) | 167 (24.3) |  |
| Black | 61 (23.7) | 169 (24.6) |  |
| Brown (mixed) | 133 (51.8) | 348 (50.6) |  |
| Other | 5 (2) | 4 (0.5) |  |
| Schooling ^a^ |  |  |  |
| < 10 years | 156 (60.7) | 362 (52.6) | <0.001 |
| ≥ 10 years | 84 (32.7) | 325 (47.2) |  |
| Preferred not to answer | 17 (6.6) | 1 (0.1) |  |
| Employment status ^a^ |  |  | 0.40 |
| Formally employee | 49 (19.1) | 152 (22.1) |  |
| Informally employee | 27 (10.5) | 60 (8.7) |  |
| Freelance work | 18 (7.0) | 56 (8.1) |  |
| Unemployed | 90 (35.0) | 260 (37.8) |  |
| Retired | 73 (28.4) | 159 (23.1) |  |
| Other/ preferred not to answer | 0 (0) | 1 (0.1) |  |
| **Clinical features** ^a^ |  |  |  |
| Type-2 diabetes | 43 (16.7) | 121 (17.6) | 0.76 |
| Blood hypertension | 114 (44.4) | 289 (42.0) | 0.52 |
| HIV infection | 2 (0.8) | 7 (1.0) | 0.74 |
| **Self-reported exposures to HCV risk factors** ^a^ | | | |
| Previous blood transfusion | 23 (8.9) | 42 (6.1) | 0.31 |
| Formal or current inject drug use | 6 (2.3) | 7 (1.0) | 0.078 |
| Tattoo or piercing | 66 (25.7) | 187 (27.2) | 0.740 |
| **Sexual behaviour in the last 6 months** ^a^ | | | |
| Sex intercourse in the last 6 months | 141 (54.9%) | 403 (58.6%) | 0.48 |
| Condomless sex ^§^ |  |  | 0.068 |
| Yes, in all sex intercourses | 114 (80.8) | 297 (73.7) |  |
| Yes, but not in all sex intercourses | 15 (10.6) | 45 (11.2) |  |
| No | 12 (8.6) | 61 (15.1) |  |
| Homosexual sex intercourse ^§^ | 4 (2.8) | 19 (4.7) | 0.34 |
| Number of sex partners ^§^ |  |  | 0.67 |
| 1-2 partners | 137 (97.2) | 385 (95.5) |  |
| 3-5 partners | 4 (2.8) | 14 (3.5) |  |
| ≥ 6 partners | 0 (0) | 4 (1) |  |

Data expressed as n (%) a or median (IQR) b . Groups were compared using Chi^2^ test for proportions and Mann-Whitney for medians. § proportion of those who had sex intercourse in the last 6 months

**Supplementary Table 2.** Comparison of observed errors and assistance provided between participants who did and who did not read instructions for use and saw the step-by-step video before HCV self-testing

|  | | **People who did not read IFU or saw step-by-step video (n=159)** | **People who read IFU and saw step-by-step video (n=526)** | **p-value** |
| --- | --- | --- | --- | --- |
| **Errors observed at each step of HCV self-testing** | | | | |
| Correct opening the test package | No | 7 (4.4) | 23 (4.4) | 0.99 |
|  | Yes | 152 (95.6) | 503 (95.6) |  |
| Correct organizing the material on the table for testing | No | 11 (6.9) | 26 (4.9) | 0.33 |
|  | Yes | 148 (93.1) | 500 (95.1) |  |
| Correct placing the tube into the stand | No | 18 (11.3) | 41 (7.8) | 0.16 |
|  | Yes | 141 (88.7) | 485 (92.2) |  |
| Correct manipulation to collect oral fluid | No | 62 (39.0) | 163 (31.0) | 0.06 |
|  | Yes | 97 (61.0) | 363 (69.0) |  |
| Correct placing of the test device in the test tube | No | 20 (12.6) | 83 (15.8) | 0.32 |
|  | Yes | 139 (87.4) | 443 (84.2) |  |
| Correct timekeeping for reading results | No | 19 (11.9) | 29 (5.5) | 0.005 |
|  | Yes | 140 (88.1) | 497 (94.5) |  |
| **Assistance provided during HCV self-testing** | | | | |
| Assistance provided for at least one step | No | 111 (69.8) | 330 (62.7) | 0.10 |
|  | Yes | 48 (30.2) | 196 (37.3) |  |

Data expressed as n (%). Groups were compared using Chi^2^ test. IFU, instructions for use
